# Investigation of the relationship between the changes in vaginal microecological enzymes and human papillomavirus (HPV) infection

**DOI:** 10.1097/MD.0000000000037068

**Published:** 2024-02-09

**Authors:** Jiawei Li, Li Jiang, Chunhua Wang, Jin Meng, Huifang Wang, Haihong Jin

**Affiliations:** a Department of Gynecology, The First Hospital of Qinhuangdao, Qinhuangdao, Hebei, P.R. China; b Department of Inspection Center, The First Hospital of Qinhuangdao, Qinhuangdao, Hebei, P.R. China.

**Keywords:** acetylglucosaminidase, enzymes, human papillomavirus, prolyl aminopeptidase, vaginal microecology

## Abstract

This study aims to investigate the relationship between the human papillomavirus (HPV) infection and the altered vaginal microecological environment of patients. Initially, HPV genotyping and microecological detection were performed in 1281 subjects in the Department of Obstetrics and Gynecology of The First Hospital of Qinhuangdao (Qinhuangdao, China). The relationship between the enzymes of vaginal microecology, that is, proline aminopeptidase and acetylglucosaminidase, and vaginal inflammatory diseases, as well as the prognosis of HPV infection, was analyzed. The experimental findings indicated a close relationship between the expression of positive prolyl aminopeptidase and trichomonas vaginitis, as well as bacterial vaginitis. In addition, the expression of acetylglucosaminidase is closely associated with trichomonas vaginitis and vulvovaginal candidiasis. Furthermore, the observations indicated that positive prolyl aminopeptidase and acetylglucosaminidase could increase the risk of various subtypes of HPV infection in patients. The receiver operating characteristic curve analysis presented that the expression of prolyl aminopeptidase and acetylglucosaminidase could offer exceptional diagnostic efficacy, indicating their association with persistent HPV infection. In summary, our results highlighted that the expression of positive prolyl aminopeptidase and acetylglucosaminidase in the vaginal microecology could be substantially correlated to the occurrence and the development of vaginal inflammatory diseases, as well as the outcome and the risk of persistent HPV infection.

## 1. Introduction

According to global statistics, human papillomavirus (HPV)-based infections have emerged as one of the serious ailments, accounting for over 600,000 cases of cancer annually.^[1]^ The predominant cause of HPV infection in most women is sexual intercourse with the HPV-infected individuals. Although the inert immune system in the body dispels the most transient type of HPV infection, the persistent type of HPV infection often leads to the integration of viral genes into the host genome, causing HPV-related cervical cancer.^[2,3]^ Noticeably, several reports demonstrated that various kinds of cervical cancers exhibited an underlying relationship with the HPV infection.^[4,5]^ In this context, the high-risk types of HPV, including HPV16 and HPV18, often result in cervical cancer in women.^[6–8]^

Previous studies indicated that the predominant factors, such as microecology, epithelial surface integrity, and immune regulation, played crucial roles in the HPV progression toward cervical cancer. Nevertheless, the functional composition of local microbiota and its changes with the infection status and disease progression remain to be comprehensively explored.^[9]^ In addition to the functional composition of local microbiota, several attributes act as essential indicators for the clinical evaluation of vaginal microecology, such as vaginal pH, hydrogen peroxide (H_2_O_2_), vaginal cleanliness, β-glucosidase, coagulase, neuraminidase, proline aminopeptidase, acetylglucosamine glycosidase, and leukocyte esterase levels.^[10–12]^ Noticeably, these essential factors in terms of vaginal acid-base balance, lactic acid bacteria function, microbial metabolites, and inflammatory reactions are collectively referred to as vaginal microecological environment. Accordingly, an increase in the vaginal pH could increase the risk of HPV infection by 30%.^[13]^ In an instance, it was demonstrated that the optimal levels of H_2_O_2_ could catalyze hypochlorite production by peroxidase, thereby preventing the colonization and growth of the HPV virus in the cervical epithelial cells.^[14]^ The poor cleanliness of the vagina might be related to the development of bacterial vaginitis (BV)^[15]^ and aerobic vaginitis.^[16]^ In an instance, a case-control study demonstrated that an abnormal rate of neuraminidase, a protein on virion for immunity and lethality, in patients with BV was significantly higher than in patients with non-BV.^[17]^ Patients with recurrent vaginal candidiasis and BV showed higher leukocyte esterase activity than the normal women of the control treatment group.^[18]^

Several enzymes in the vagina, such as proline aminopeptidase and acetylglucosamine glycosidase, play crucial roles in the overall health of vaginal microecology. On the one hand, the expression of positive proline aminopeptidase designates the existence of various vaginal inflammatory diseases, such as BV and trichomonas vaginitis (TV). On the other hand, the primary function of the expression of acetylglucosamine glycosidase indicates the infection of candida albicans and trichomonas vaginalis. Acetylglucosamine glycosidase is a specific enzyme secreted by candida albicans and trichomonas vaginalis, which is not confined to normal vaginal secretions. These findings of clinical measures can be further developed to prevent and treat cervical lesions by detecting changes in the vaginal microbiota and enzymatic secretions of patients.

Motivated by these considerations, this study aims to systematically explore the correlation between the expression levels of enzymes (proline aminopeptidase and acetylglucosamine glycosidase) and vaginal inflammatory diseases (vaginitis) that lead to the risk of HPV infection. The correlation highlights the impact of changes in the vaginal microbiota and enzymatic levels on the occurrence and development of HPV infection. These findings exploring the correlation between the enzymes and HPV infection will undoubtedly indicate the potential solution towards reducing the incidence and mortality rates, improving prognosis, and providing possibilities for early detection, diagnosis, and treatment of cervical cancer.

## 2. Materials and methods

### 2.1. Subjects

The subjects (n = 1281) for this study were recruited selectively from women who visited the Department of Obstetrics and Gynecology of our hospital from January 2020 to June 2022. The age range of the subjects was between 18 and 64 years, with an average age of 34.2 ± 4.33 years. The inclusion criteria for selecting the eligible subjects were set as follows: Patients with a history of sexual activity within the past 3 days; patients who had a history of cervical lesion treatment or chemotherapy, no severe immune system diseases, and no sexually transmitted diseases. To this end, the exclusion criteria were set as follows: patients who had flushed the vagina within 48 hours, patients who were prescribed medication or sexual intercourse within the past 3 days, and consuming antibiotics orally within 1 month. This study was approved by the Medical Ethics Committee of the First Hospital of Qinhuangdao. All experimental studies were conducted in compliance with the relevant regulations and under the supervision and guidance of the Ethics Committee. Notably, a signed informed consent was provided by all the recruited participants.

### 2.2. Sample collection

Initially, the recruited subject was directed to take a bladder lithotomy position on the examination bed. Further, a sterile vaginal speculum lubricated with the physiological saline was gently placed in the vagina to clear the cervix. Then, the sample was collected by brushing 2 cm away from the cervical canal and swapping 3 to 5 turns around the outer opening of the cervix. After collecting the specimen, the speculator was immediately placed in the preservation solution and tightened the sample bottle cap to avoid any bacterial infection.

### 2.3. Evaluations

#### 2.3.1. Vaginal microbiota detection.

As mentioned earlier, the vaginal secretion was collected by inserting the speculum from one-third of the lateral vaginal wall. Further, the vaginal secretion was dispersed on the clean glass slides and observed under a microscope for trichomonas and hyphae. Further, the vaginal pH value was determined using a color bar indicator, in which the pH value of ≤4.5 was determined as normal, as the nominal vaginal pH. Contrarily, the pH value of >4.5 was represented as abnormal. Considering the cleanliness indicators, the vaginal cleanliness of I and II grades were denoted as normal vaginal cleanliness, while the III and IV grades were indicated as abnormal vaginal cleanliness. Eventually, the nominal microecology of the vagina was presented as dominant lactic acid bacteria, negative H_2_O_2_ levels (−), normal white blood cell esterase of negative (−) or (±), and negative sialidase, proline aminopeptidase, acetylglucosamine glycosidase, as well as β-glucuronidase levels (−).

#### 2.3.2. HPV genotyping.

Polymerase chain reaction-reverse dot blot hybridization was employed to determine the expression of the HPV infection. A total of 25 HPV subtypes were detected. Among them, 8 subtypes (6, 11, 40, 42, 43, 44, 81, and 83) were determined as low-risk, and the rest 17 of them were presented as high-risk, including 16, 18, 26, 31, 33, 35, 39, 45, 51, 52, 53, 56, 58, 59, 66, 68, and 73.

### 2.4. Statistical analysis

The collected data were statistically presented in terms of frequency (percentage, n%). The data between different groups were compared using the chi-square test. The diagnostic efficacy of the model for diseases was evaluated using the receiver operating characteristic (ROC) curve analysis of the subjects. The data were processed using the SPSS statistical software package (v23.0, IBM Corporation, Armonk, USA), considering the *P* value of <.05 statistically significant.

## 3. Results

### 3.1. Relationship between vaginal microecological enzymes and vaginitis

In the study, the recruited subjects (n = 1281 women) showed the typically manifested symptoms of various vaginal inflammatory diseases, including 10 cases of TV, 29 cases of BV, and 63 cases of vulvovaginal candidiasis (VVC). Further, the observations indicated that 4 cases suffering from TV and 7 cases from BV possessed positive proline aminopeptidase, accounting for 3.54% and 6.19%, respectively. These positive proline aminopeptidases were significantly higher than those with the negative proline aminopeptidase (*P* < .05), indicating the incidence rate of positive proline aminopeptidase in TV and BV patients. To this end, 8 cases of TV and 42 cases of VVC patients showed the expression of positive acetylglucosamine glycosidase, accounting for the incidence rates of 6.50% and 34.15%, respectively. Noticeably, the expression levels of positive acetylglucosamine glycosidase were significantly (*P* < .05) higher than those with negative acetylglucosamine glycosidase. The detailed summary of expression levels of different enzymes and their relationship with vaginitis, along with the significance values, is presented in Table 1.

**Table 1:** Association between vaginal microecological enzymes and 3 types of vaginitis.

Functional indicators	Prolyl aminopeptidase		*P* value	Acetylglucosaminidase		*P* value
	Positive	Negative		Positive	Negative	
TV						
Yes	4 (3.54)	6 (0.51)	.008	8 (6.50)	2 (0.17)	.000
No	109 (96.46)	1162 (99.49)		115 (93.50)	1156 (99.83)	
BV						
Yes	7 (6.19)	22 (1.88)	.01	1 (0.81)	28 (2.42)	.353
No	106 (93.81)	1146 (98.12)		122 (99.19)	1130 (97.58)	
VVC						
Yes	10 (8.85)	53 (4.54)	.426	42 (34.15)	21 (1.81)	.000
No	103 (91.15)	1115 (95.46)		81 (65.85)	1137 (98.19)	

BV = bacterial vaginitis, TV = trichomonas vaginitis, VVC = vulvovaginal candidiasis.

### 3.2. Correlation between proline aminopeptidase and HPV infection

Prior to establishing the correlation between proline aminopeptidase and HPV infection, the HPV infection status of the recruited subjects was initially determined. The HPV testing analysis indicated that 509 cases of the recruited subjects were HPV-positive, with an infection rate of 39.73%. Moreover, the HPV phenotyping indicated 304 cases of HPV16-positive, 209 cases of HPV18-positive, and 212 cases of HPV other types. Among them, 80 (70.79%), 72 (63.72%), 68 (60.18%), and 61 (53.98%) cases of patients showed the expression of positive proline aminopeptidase, respectively (Table 2). Noticeably, a significantly higher proportion of cases with proline aminopeptidase in patients with positive HPV subtypes infection was observed than in patients with negative HPV subtypes (*P *< .05). Contrarily, the proportion of cases with negative proline aminopeptidase in patients with positive HPV subtypes infection was lower than in patients with negative HPV subtypes. These findings suggested the positive correlation between the detection rate of HPV subtypes infection in patients and the expression of positive proline aminopeptidase in their vaginal microecology.

**Table 2:** Association between prolyl aminopeptidase and HPV infection.

HPV infection	Prolyl aminopeptidase positive (n = 113) %	Prolyl aminopeptidase negative (n = 1168) %	*χ* ^2^	*P* value
Positive			6.237	.011
Yes	80 (70.79)	429 (36.73)		
No	33 (29.21)	739 (63.27)		
HPV16			10.392	<.001
Yes	72 (63.72)	232 (19.86)		
No	41 (36.28)	936 (80.14)		
HPV18			9.927	.002
Yes	68 (60.18)	141 (12.07)		
No	45 (39.82)	1027 (87.93)		
Other HPV types			14.927	<.001
Yes	61 (53.98)	151 (12.93)		
No	52 (46.02)	1017 (87.07)		

HPV = human papillomavirus.

### 3.3. Relationship between acetylglucosidase and HPV infection

Similar to proline aminopeptidase, the acetylglucosidase-positive cases were further determined and correlated with the risk of HPV infection. Among the positive HPV cases, the acetylglucosidase-positive cases were 91 (73.98%), 84 (68.29%), 73 (59.35%), and 67 (54.47%) for HPV-positive, HPV16-positive, HPV18-positive, and other subtypes of HPV-positive patients, respectively. These results indicated that a significantly higher number of patients expressed positive acetylglucosidase than the proportion of HPV subtype negative patients (*P* < .05, Table 3). Similar to positive proline aminopeptidase, these findings suggested that the detection rate of HPV subtype-positive patients could be correlated with the expression of acetyl glucosidase levels in their vaginal microecology.

**Table 3:** Association between acetylglucosaminidase and HPV infection.

HPV infection	Acetylglucosaminidase positive (n = 123)	Acetylglucosaminidase negative (n = 1158)	*χ* ^2^	*P* value
Positive			15.927	<.001
Yes	91 (73.98)	418 (36.10)		
No	32 (26.02)	740 (63.90)		
HPV16			11.872	<.001
Yes	84 (68.29)	220 (19.00)		
No	39 (31.71)	938 (81.00)		
HPV18			6.931	.008
Yes	73 (59.35)	136 (11.74)		
No	50 (40.65)	1022 (88.26)		
Other HPV types			10.038	<.001
Yes	67 (54.47)	145 (12.52)		
No	56 (45.53)	1013 (87.48)		

HPV = human papillomavirus.

### 3.4. Association between the microbial enzymes of different HPV infection types

Further, the correlation between the microbial enzymes of different HPV infection types was demonstrated. Initially, we randomly distributed the HPV-positive patients into 3 different groups based on HPV infection status, that is, HPV16/18, other types of high-risk HPV (HR-HPV), and low-risk HPV (LR-HPV). The statistics of distribution included 297 HPV16/18-positive patients, 138 HR-HPV-positive patients, and 74 LR-HPV-positive patients. Noticeably, it was observed that the positivity infection rate of HPV16/18 in patients with β-glucuronidase-positive, sialidase-positive, leukocyte esterase-positive, and hydrogen peroxide abnormality was significantly higher compared to HR-HPV positive patients. Comparatively, the positivity rate of other HR-HPV-positive patients was significantly higher than that of LR-HPV-positive patients regarding the expression levels of notified enzymes (*P* < .05, Table 4).

**Table 4:** The vaginal microecological enzymes of the 3 groups of HPV-infected subjects.

Functional indicators	HPV 16/18 (n = 297)		HR-HPV (n = 138)		LR-HPV (n = 74)		*P* value
	Number of cases	Composition ratio (%)	Number of cases	Composition ratio (%)	Number of cases	Composition ratio (%)	
β-Glucuronidase-positive	186	62.63	72	52.17	35	47.30	<.05
β-Glucuronidase-negative	111	37.37	66	47.83	39	52.70	
Sialidase-positive	227	76.43	85	61.59	41	55.41	<.05
Sialidase-negative	70	23.57	53	38.41	33	44.59	
Leukocyte esterase-positive	233	78.45	84	60.87	40	54.05	<.05
Leukocyte esterase-negative	64	21.55	54	39.13	34	45.95	
Hydrogen peroxide - normal	189	63.64	98	71.01	56	75.68	<.05
Hydrogen peroxide - deficiency	108	36.36	40	28.99	15	24.32	

HPV = human papillomavirus.

### 3.5. Diagnostic potential of enzymes for HPV infection

**Figure 1.**
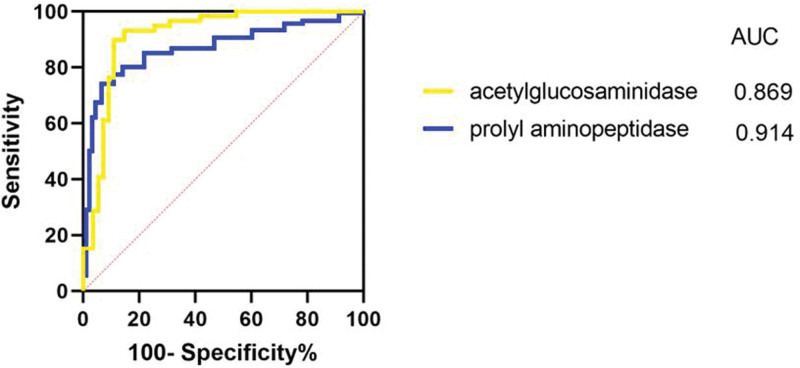
The receiver operating characteristic (ROC) curve efficiently analyzes the vaginal microecological enzymes and HPV infection.

Further, the relationship between the expression levels of different enzymes and HPV infection status motivated us to explore the diagnostic potential of enzymes against HPV infection. To demonstrate this aspect, the ROC curve was plotted using the values of vaginal proline aminopeptidase and N-acetylglucosaminidase expression levels and predicted their probability values for both variables. After analysis, the area under the curve values of the predicted probabilities for prolidase and N-acetylglucosaminidase were 0.914 and 0.869, respectively (Fig. 1). Together, these findings indicated that proline aminopeptidase and acetylglucosaminidase could show excellent diagnostic potency to determine the HPV infection.

### 3.6. Relationship between microecological enzymes and the persistence of HPV infection

It was observed from the data that the proline aminopeptidase-positive cases were 54.78% of 115 patients with HPV persistent infection, which was significantly higher than the proportion of patients with HPV transient infection (20.56%) (*P* < .05). In addition, 53.04% of HPV persistent infection patients expressed positive acetylglucosaminidase, which was significantly higher than the proportion (21.57%) of positive acetylglucosaminidase in the transient HPV infections (*P* < .05). Together, these findings of incidence rates for both proline aminopeptidase and acetylglucosaminidase enzymes could be undoubtedly associated with persistent HPV infection (Table 5).

**Table 5:** The vaginal microecological enzymes of the HPV infection groups of research subjects.

Functional indicators	Transient HPV infection (n = 394)		Persistent HPV infection (n = 115)		*χ* ^2^	*P* value
	Number of cases	Composition ratio (%)	Number of cases	Composition ratio (%)		
Prolyl aminopeptidase positive (n = 144)	81	20.56	63	54.78	16.789	<.001
Prolyl aminopeptidase negative (n = 1137)	313	79.44	52	45.22		
Acetylglucosaminidase positive (n = 146)	85	21.57	61	53.04	17.832	<.001
Acetylglucosaminidase negative (n = 1135)	309	78.43	54	46.96		

HPV = human papillomavirus.

## 4. Discussion

Vaginitis has emerged as one of the most prevalent gynecological inflammatory diseases in clinics, including several subtypes of TV, BV, CV, VVC, and nonspecific vulvovaginitis. The predominant etiological factors include decreased immunity, altered menstrual cycle, changes in the sexual hormone levels, and long-term administration of hormones, among others. Typically, the routine biochemical examination of numerous vaginal secretions is the foremost method for diagnosing different vaginitis in clinical practice. However, this conventional diagnostic approach suffers from a major limitation of a low detection rate. To address this limitation, several efforts have been dedicated to gradually replacing it with the 5-fold vaginitis test for detecting various molecules and enzymes. This 5-fold vaginitis test kit detects various molecules and enzymes that are specifically associated with a pathogenic bacterial infection, such as H_2_O_2_, leukocyte esterase, sialidase, proline aminopeptidase, and acetylglucosaminidase. Noticeably, this kit offers specific attributes, such as easy operation, precision, and rapidity, to precisely determine the subtype of vaginitis.

Several kinds of vaginal microbiome communities, that is, more than 100 species, have been identified in the women vaginal microenvironment. These microbiome communities often exist in antagonistic and interdependent relationships, maintaining a homeostatic steady state in the vaginal microenvironment. Nonetheless, the microecological balance of the microbiome is often influenced by various features, including endocrine secretion, local immunity, and the anatomy of the reproductive system. In the case of imbalance, the vaginal microecological system triggers multiple inflammatory diseases. Accordingly, this current study demonstrated that various subtypes of vaginitis were closely related to the vaginal microecology towards the risk of HPV infection. Importantly, the patients with TV and BV showed significantly higher positive proline aminopeptidase detection rates in the vaginal secretions over the negative proline aminopeptidase. The probability of infecting with TV and *Pseudomonas*-based vulvovaginal vaginitis was significantly higher with the increased detection rate of positive acetylglucosaminidase in the vaginal secretions of the patients.

In addition to vaginitis, the vaginal microecology is closely related to the persistence and clearance of HPV infection, as well as the cervical squamous epithelial lesions. Previous studies indicated that *Lactobacillus* was the dominant bacterium, significantly balancing the ecological system and shielding the female lower reproductive tract health.^[19]^ In this framework, various species of lactobacilli in the vaginal microbiota included *L crispatus, L gasseri*, and *L jensenii*.^[20]^ Several reports demonstrated that women with a non-*Lactobacillus* predominance in the vagina displayed a higher risk of infection with HR-HPVs than women with an *L. crispatus* predominance.^[21,22]^ In a case, Lee and colleagues^[11]^ observed that the proportion of *Lactobacillus* was lower in the HPV(+) patients compared to the control treatment group. On the one hand, lactobacilli maintained the weak acidic environment of the vagina through the production of lactic acid. On the other hand, a large number of lactic acid bacteria could reduce and inhibit the growth of some conditional pathogenic bacteria, protecting the lower genital tract from infection.^[23,24]^ In 1992, a study revealed that the laboratory culture could substantially manifest the hallmark “abnormal vaginal microecology” in CIN,^[25]^ which was further confirmed in their subsequent studies.

As specified earlier, the disruption of the vagina microecology could diminish the defense mechanism against various pathogenic microbiome communities, indicating the close association between vaginal microecology with the onset of vaginal infection. Accordingly, analyzing vaginal microecology indicators is expected to be beneficial for understanding, evaluating, and treating different vaginal infections, including vaginitis. In this study, we demonstrated the role of various enzymes (prolyl aminopeptidase and acetylglucosaminidase) in vaginitis and their influence on the risk of HPV infection. It was observed that the prevalence rates of various subtypes of HPV infections were significantly higher in the prolyl aminopeptidase-positive patients than in prolyl aminopeptidase-negative patients. Similarly, the prevalence rates of HPV infections with each subtype were significantly higher in the acetylglucosaminidase-positive patients than in acetylglucosaminidase-negative patients. These findings indicated that HPV infection and changes in the expression levels of enzymes in the vaginal microecological system were substantially related. The proline aminopeptidase-positive and acetylglucosaminidase-positive levels were correlated with the high-risk HPV infection. Moreover, other important factors in the vaginal microecology and their role were observed. Along this line, we analyzed the vaginal microecology by dividing the HPV-infected patients into the HPV16/18 group with the prevalence of infection. In addition to the proline aminopeptidase-positive and acetylglucosaminidase-positive, the changes in expression of β-glucuronidase, sialidase, and leukocyte esterase enzymes, as well as hydrogen peroxide levels were observed. Interestingly, the higher the risk of HPV, the more pronounced its impact on vaginal microecology. Previous reports demonstrated that the HR-HPV infection could interfere with the gene regulatory function, disrupt genomic stability, and quickly induce malignant lesions. The prolonged infection time could promote the cervical lesions to progress to a high grade and shorten the time of cervical precancerous lesions to cancer.^[26,27]^ Therefore, early prediction of the persistence of HPV infection is of great clinical significance.

Eventually, the ROC curve was plotted using the values of vaginal microecological enzymes, that is, proline aminopeptidase and acetylglucosaminidase, and their combined predictive probability values as test variables. It was observed that the expression levels of proline aminopeptidase and acetylglucosaminidase possessed excellent diagnostic efficacy for HPV infection. Despite the success in exploring the relationship between vaginal microecological enzymes and HPV infection and its further role in HPV diagnosis, the current study suffers from certain limitations. Firstly, this study was substantially focused on exploring the relationship between vaginal microecology, inflammatory vaginosis, and the occurrence, as well as the development of HPV infection from the perspective of proline aminopeptidase and N-acetylglucosaminidase. In contrast, it is required to explore the altered microecological factors and other involved enzymes for the development of HPV infection. Secondly, our sample size was not large enough considering the important HPV genera. Moreover, the HPV grouping was insignificant, which might result in biased findings. The follow-up study is required to consider more HPV-infected cases, combining the latest research progress and scientific rational design grouping.

## 5. Conclusion

In summary, the altered vaginal microecology could be correlated with the risk of HPV infection. Moreover, the incidence rates of positive proline aminopeptidase and acetylglucosaminidase were correlated with the inflammatory diseases in the vagina, which could be related to the occurrence, development, and outcome of HPV infection. These enzymes guiding early detection could substantially target the prevention of HPV infection and reduce the risk of HPV persistent infection. Together, these findings provide a greater possibility for early detection of cervical cancer, early diagnosis, and early treatment, reducing the incidence and mortality of cervical cancer, as well as improving the prognosis effect.

## Author contributions

**Conceptualization:** Haihong Jin.

**Data curation:** Jin Meng.

**Formal analysis:** Li Jiang.

**Methodology:** Li Jiang, Chunhua Wang, Huifang Wang.

**Project administration:** Huifang Wang.

**Software:** Chunhua Wang.

**Supervision:** Chunhua Wang, Jin Meng.

**Validation:** Jin Meng.

**Writing – original draft:** Jiawei Li.

**Writing – review & editing:** Haihong Jin.
